# The bodies of *dpy-10(e128)* are twice as stiff as wild type

**DOI:** 10.17912/ecsm-mp67

**Published:** 2018-08-06

**Authors:** Sylvia Fechner, Frédéric Loizeau, Adam L. Nekimken, Beth L. Pruitt, Miriam B. Goodman

**Affiliations:** 1 Molecular and Cellular Physiology, Stanford School of Medicine, Stanford; 2 Departments of Bioengineering and Mechanical Engineering, Stanford; 3 Departments of Mechanical Engineering and Biomolecular Science and Engineering, University of California, Santa Barbara

**Figure 1.**
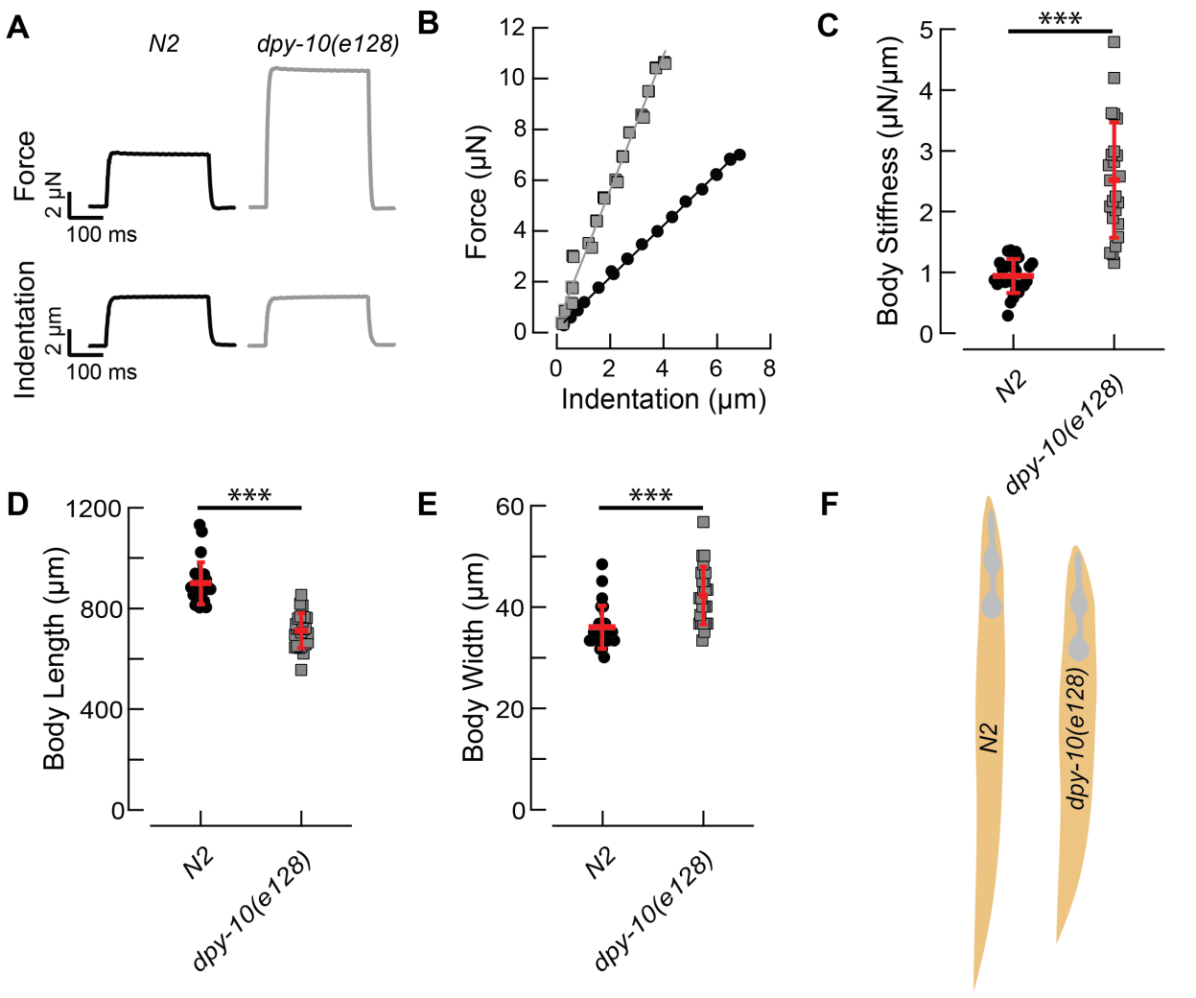
Panel A shows the force and indentation produced in response to a mechanical load applied by pushing a glass bead mounted on a self-sensing cantilever into the worm’s body. Panel B shows a set of 45 force and indentation pulses to determine the overall relationship between force and indentation for a *dpy-10* (gray) and a wild-type (N2, black) L4 or young adult hermaphrodite. The force-indentation plots were fitted with a line where the slope is taken as a measure of body stiffness. Panels C-E show the measurements derived from more than 20 animals and the red bar indicates mean ± standard deviation.

## Description

DPY-10 is a collagen protein in the nematode’s cuticle. Mutations in the *dpy-10* gene induce various morphological changes that lead to animals with a dumpy (Dpy) or roller (Rol) phenotype (Levy, Yang & Kramer, 1993). Here, we asked how such mutations affect body stiffness by comparing force-indentation curves in *dpy-10(e128)* and wild type worms. On average, *dpy-10(e128)* worms have a steeper force-indentation relationship, leading to a higher body stiffness (Fig 1C). Average stiffness values were 2.5 ± 0.9 N/m, SD, (n = 24) for *dpy-10(e128)* and 0.9 ± 0.3 N/m, SD, (n = 25) for wild type (N2) hermaphrodites. Qualitatively, we observed that some of the *dpy-10(e128)* worms had a disrupted cuticle and their bodies spontaneously eviscerated after being immobilized to an agarose pad. Finally, we confirm the finding that *dpy-10(e128)* worms are shorter (Fig 1D) and fatter (Fig 1E) than wild type worms with an average body length of 712 ± 68 µm (SD, n = 24) compared to wild type N2 worms 900 ± 68 µm (SD, n = 25) and an average body width at the pharynx of 42 ± 5.6 µm (SD, n = 24) compared to wild type N2 worms 36 ± 4 µm (SD, n = 25) (Levy, Yang & Kramer, 1993). Figure 1F shows body shape and size of the two genotypes (drawn to scale).

From these results, we conclude that the bodies of *dpy-10(e128)* are twice as stiff as wild type: to reach the same indentation, a much higher force was necessary to be applied to *dpy-10(e128)* than to wild type worms (Fig 1A). Prior measurements of body stiffness with the same method revealed that *dpy-5(e61)* worms are softer than wild type worms (Park et al. 2007). Thus, body shape does not predict body stiffness. Why is one Dpy animal softer than wild-type and another one stiffer? We propose that *dpy-10* is stiffer than wild type because it has an increased internal glycerol concentration and increased pressure*,* that is absent from *dpy-5(e61)* (Wheeler and Thomas 2006).

## Methods

*Preparation of worms:* Age-synchronized *C. elegans* nematodes were grown on standard OP50 growth plates at 20–23 °C. Late L4 or young adult hermaphrodites were immobilized on 8% agarose pads using WormGlu (GluStitch). During the experiments the worms were continuously superfused with extracellular saline.

*Force-Indentation measurements:* The measurements were performed with the FALCON system described in Eastwood et al, 2015. In brief, we used piezoresistive cantilevers to deform the worm’s cuticle and to read out the respected indentation and force. Measurements were performed in displacement clamp. The cantilever had a spring constant *kc* of 1.3 N/m and a resonant frequency of 12.2 kHz. A glass microsphere (10 ± 1-μm diameter) was adhered to the cantilever tip. Cantilevers were mounted to a piezoelectric actuator. Because the device was placed directly above the worm’s surface before each experiment, sample indentation depth (*z*) was calculated as *z* = *x*t – *x*c,where *x*t is the total actuator displacement as measured by the built-in sensor on the actuator and *x*c is the cantilever deflection as measured by the piezoresistor, as described (Park et al., 2007; Eastwood et al., 2015). Force was calculated as *F*= *x*t*kc. For each animal, we applied 15 displacement steps (0.5 – 12.5µm) in three replicates. The cantilever was positioned 154 ± 37 μm behind the pharynx. Data acquisition occurred with HEKA software and a sampling frequency of 10 kHz. Analysis was performed in Matlab.

## Reagents

CB128: *dpy-10(e128); N2. Strains available at the CGC.*Extracellular saline: in mM: NaCl (145), KCl (5), MgCl2 (5), CaCl2 (1), Hepes (10), adjusted to pH 7.2 with NaOH. 8% agarose (wt/vol) dissolved in extracellular saline, pH adjusted to 8.01 with NaOH.
